# Differential mRNA Expression and Glucocorticoid-Mediated Regulation of TRPM6 and TRPM7 in the Heart and Kidney throughout Murine Pregnancy and Development

**DOI:** 10.1371/journal.pone.0117978

**Published:** 2015-02-18

**Authors:** James S. M. Cuffe, Sarah Steane, Karen M. Moritz, Tamara M. Paravicini

**Affiliations:** School of Biomedical Sciences, The University of Queensland, Brisbane, Australia; University of Rennes-1, FRANCE

## Abstract

The transient receptor potential (TRP) channels TRPM6 and TRPM7 are critically involved in maintaining whole body and cellular Mg^2+^ homeostasis and ensuring the normal function of organs such as the heart and kidney. However, we do not know how the expression of TRPM6 and TPRM7 in these organs changes throughout fetal development and adult life, and whether this expression can be hormonally regulated. This study determined the ontogeny of TRPM6 and TRPM7 mRNA expression from mid-gestation through to adulthood in the mouse. In a second series of experiments, we examined how maternal administration of the glucocorticoids corticosterone and dexamethasone between embryonic days 12.5–15 affected TRPM6 and TRPM7 channel mRNA expression in the mother and fetus. Whilst renal TRPM7 expression was relatively constant throughout development, renal TRPM6 expression was markedly upregulated after birth. In contrast, cardiac TRPM7 expression was 2–4 fold higher in the fetus than in the adult. Surprisingly, TRPM6 expression was detected in the fetal heart (qPCR and *in situ* hybridization). Glucocorticoid administration during gestation increased fetal cardiac expression of both channels without affecting renal expression. In contrast, in the dam renal TRPM6 and TRPM7 expression was increased by glucocorticoids with no change in the cardiac channel expression. These data suggest that TRPM6 and TRPM7 channels are important in organogenesis, and that elevated maternal glucocorticoid levels can alter the expression of these channels. This suggests that perturbations in hormonal regulatory systems during pregnancy may adversely impact upon normal fetal development, at least in part by altering expression of TRPM channels.

## Introduction

Magnesium is an abundant intracellular cation that is crucial for many fundamental processes involved in normal cell function, including protein synthesis, DNA replication and energy metabolism [1]. In light of this, serum magnesium levels are kept tightly controlled, with overall body magnesium homeostasis reflecting the balance between intestinal absorption and renal excretion [2]. Intracellular magnesium concentrations are also maintained within a narrow range, however until recently the molecular mechanisms regulating magnesium transport at the cellular level were poorly understood.

Two members of the transient receptor potential (TRP) channel superfamily, TRPM6 and TRPM7, have now been identified as critical regulators of magnesium homeostasis. Genetic analyses of patients with autosomal-recessive hypomagnesemia and secondary hypocalcemia (HSH) indicate that TRPM6 is an essential gene for magnesium homeostasis. Studies of HSH patients by two independent research groups identified multiple mutations in TRPM6 that caused abnormal renal magnesium handling and hypomagnesemia [3–5]. TRPM6 is predominantly expressed in the lung, cecum, colon and renal tubules [6]. Whilst overall magnesium homeostasis is influenced by intestinal absorption and dynamic exchange with bone, the major site of regulation is the kidney, which controls magnesium excretion to balance intestinal absorption [3]. The amount of magnesium lost in the urine is ultimately determined by how much magnesium is actively reabsorbed in the distal convoluted tubule, a process thought to be mediated (at least in part) by TRPM6 [7]. Thus, we can consider TRPM6 to be a regulator of whole body magnesium homeostasis.

In contrast, the TRPM7 channel is ubiquitously expressed [6,8], with high levels of expression seen in the heart and kidney [9]. A magnesium- and calcium-permeable ion channel with homology to TRPM6, the primary physiological role of TRPM7 appears to be the maintenance of cellular magnesium homeostasis [8,10]. TRPM7 is also critical for cell growth, as genetic deletion of TRPM7 in cultured cells prevents proliferation, an effect that can be reversed with either external magnesium supplementation or overexpression of other cellular magnesium transporters [10–13]. Like TRPM6, TRPM7 has also been shown to be critical for systemic magnesium homeostasis [14].

Both TRPM6 and TRPM7 appear to play critical roles in embryonic development. Global deletion of TRPM7 in transgenic mice is lethal, causing embryonic loss before day 7.5 of embryogenesis [15]. Mice lacking TRPM6 also show high rates of embryonic loss (often before embryonic day 12.5), and those that survived to this point exhibited neural tube defects [16]. However, despite these observations, the regulation of TRPM6 and TRPM7 throughout development, and the potential roles of these channels in fetal organ development, are yet to be fully defined.

Multiple factors can regulate TRPM6 and TRPM7 expression in adulthood; these include altered dietary magnesium intake [6,17,18] and steroid hormones such as aldosterone and glucocorticoids [17,19]. The latter may be of particular relevance during development, as pregnant women are often exposed to either natural or synthetic glucocorticoids during gestation [20] for numerous reasons. Whilst the prevalence of glucocorticoid exposure during pregnancy is difficult to quantify, the overall incidence is likely to be high. Importantly, prenatal exposure to elevated glucocorticoid levels has been shown to disturb normal fetal growth and organ development [20]. The developing heart and kidney have been shown to be particularly vulnerable to maternal glucocorticoid exposure, which can alter development of these organs and ‘program’ disease in adult life [21,22]. Given that TRPM6 and TRPM7 are critical for normal fetal development and that glucocorticoids may affect the expression of these channels, it is possible that glucocorticoid-induced changes in fetal magnesium channel expression during gestation may alter magnesium homeostasis and thereby affect fetal development.

This leads to the two main aims of the present study. First, we aimed to characterize how the expression of TRPM6 and TRPM7 changes throughout the development of the heart and kidney, from the fetus into adulthood. The second aim was to determine whether maternal administration of glucocorticoids during mid-gestation affects the maternal and fetal expression of TRPM6 and 7 in the heart and kidney.

## Materials and Methods

### Animal studies

All animal procedures were approved by The University of Queensland Anatomical Biosciences Animal Ethics Committee (AEC approval number SBMS/355/09) and conducted in accordance with the Australian Code of Practice for the Care and Use of Animals for Scientific Purposes. Female C57Bl6/J mice were time-mated overnight at 8–10 weeks of age. Pregnancy was confirmed the following morning by the presence of a seminal plug, and this time point defined as embryonic day (E) 0.5. For ontogeny studies, dams and pups were euthanized at either E14.5 or E17.5, or allowed to deliver naturally. For animals that delivered, the offspring were euthanized at post-natal day 30 (PN30) or as adults. Following euthanasia the hearts and kidneys were collected and snap frozen in liquid nitrogen (for quantitative PCR) or fixed in 4% paraformaldehyde followed by paraffin embedding (for *in situ* hybridization).

### Glucocorticoid treatment

To determine the effects of glucocorticoids on TRPM6 and TRPM7 expression during development, pregnant females were treated with glucocorticoids for 60 hours from E12.5 as described previously [23–25]. Dams were anaesthetized using isoflurane (2%) and an osmotic minipump (model #1003D, Alzet, CA) inserted subcutaneously in the subscapular region. The osmotic pumps contained the synthetic glucocorticoid dexamethasone (Dex, dexamethasone sodium phosphate, Intervet, Australia; 1 μg/kg/hr), the endogenous rodent glucocorticoid corticosterone (Cort, Sigma-Aldrich Australia; 33 μg/kg/hr) or 0.9% saline as a control. All drugs were delivered at a rate of 1 μL/hr. Mice were then euthanized either during the infusion period at E14.5 (after ∼ 48 hours of glucocorticoid treatment) or at E17.5 (∼ 60 h after the cessation of glucocorticoid treatment) and tissues collected from the pups and dams as detailed above. These doses of glucocorticoids (delivered during this period of gestation in the mouse) are known to adversely affect fetal and placental growth, and cause alterations in the hearts and kidneys of adult offspring [23–25].

### Plasma electrolytes

Whole blood was collected by cardiac puncture from the dams at E14.5 following euthanasia. Plasma was separated by centrifugation and stored at −70°C until assay. Plasma electrolytes were measured using an automated analyzer (Cobas Integra 400 Plus, Roche Diagnostics).

### Gene expression

Gene expression of TRPM6 and TRPM7 was measured using quantitative reverse transcriptase PCR (qPCR). Total RNA was extracted from frozen heart and kidney samples using a commercially available extraction kit (RNeasy, Qiagen) according to the manufacturer’s instructions. All samples were treated with DNase to remove contaminating genomic DNA and quantified spectrophotometrically before being reverse transcribed using random primers (Invitrogen Superscript III). Taqman qPCR was performed using exon-spanning gene expression assays (Applied Biosystems) for *Trpm6* (Mm00463112_m1, assay location 1523 spanning exons 13–14) and *Trpm7* (Mm00457998_m1, assay location 1785 spanning exons 13–14). Ribosomal 18S was amplified in the same reaction tube using a VIC-labelled probe and primers (Applied Biosystems endogenous control reagents). Expression levels were analyzed using the comparative 2^-ddCt^ method [26]. Expression of the sex-specific gene *Xist* (Mm01232884_m1) was used to confirm fetal sex [24]. As there were no sex-specific expression differences in any tissue from E14.5, E17.5 or PN30 mice, the data from both sexes has been combined at these time points.

### *In situ* hybridization

To localize the distribution of *Trpm6* and *Trpm7* RNA, 10 μm sections were taken from paraffin embedded hearts and kidneys from fetal (E17.5) and adult mice. Antisense probes (679 and 613 nucleotides for TRPM6 and TRPM7 respectively) and matching sense negative control probes were designed using NCBI sequence data (*Trpm6* NM_153417; *Trpm7* NM_021450) and generated from pooled adult kidney and heart cDNA using the following primers: TRPM6 Forward primer TAA TAC GAC TCA CTA TAG GGG CCT GTC AAA GAA GAA GAG GAA; TRPM6 reverse primer AAT TAA CCC TCA CTA AAG GGG GGG AGA AAA GAC TTC ACA ATG; TRPM7 forward primer TAA TAC GAC TCA CTA TAG GGG TGG GAG AAA ACT TGA CTG ACC; TRPM7 reverse primer AAT TAA CCC TCA CTA AAG GGC TTA GCT GAA TGG CTG TGA CTG. These primers included a leading promoter sequence for either the T7 RNA polymerase (forward primers) or T3 RNA polymerase (reverse primers). 200 ng of each PCR product was used to generate both the sense and antisense DIG labelled RNA probes using either the T7 or T3 RNA polymerase. *In situ* hybridization was performed as previously described [27]. Briefly, slides were post-fixed with 4% paraformaldehyde, treated with proteinase K and acetylated before hybridization with antisense probes at 70°C overnight. Additional slides were treated in an identical manner and were hybridized with the sense probes to act as negative controls. Slides were then stained with NBT/BCIP (blue) and counterstained using nuclear fast red. Sections were scanned using an Aperio scanscope XT slide scanner (Aperio, Vista, CA).

### Statistics

Data is presented as mean ± SEM and analyzed using one-way ANOVA with either Tukey's (ontogeny) or Dunnett's (glucocorticoid treatment) post-hoc tests for multiple comparisons. All data was analyzed using GraphPad Prism 5, and *P*<0.05 was considered statistically significant.

## Results

### Renal TRPM6/7 expression

**Fig 1:**
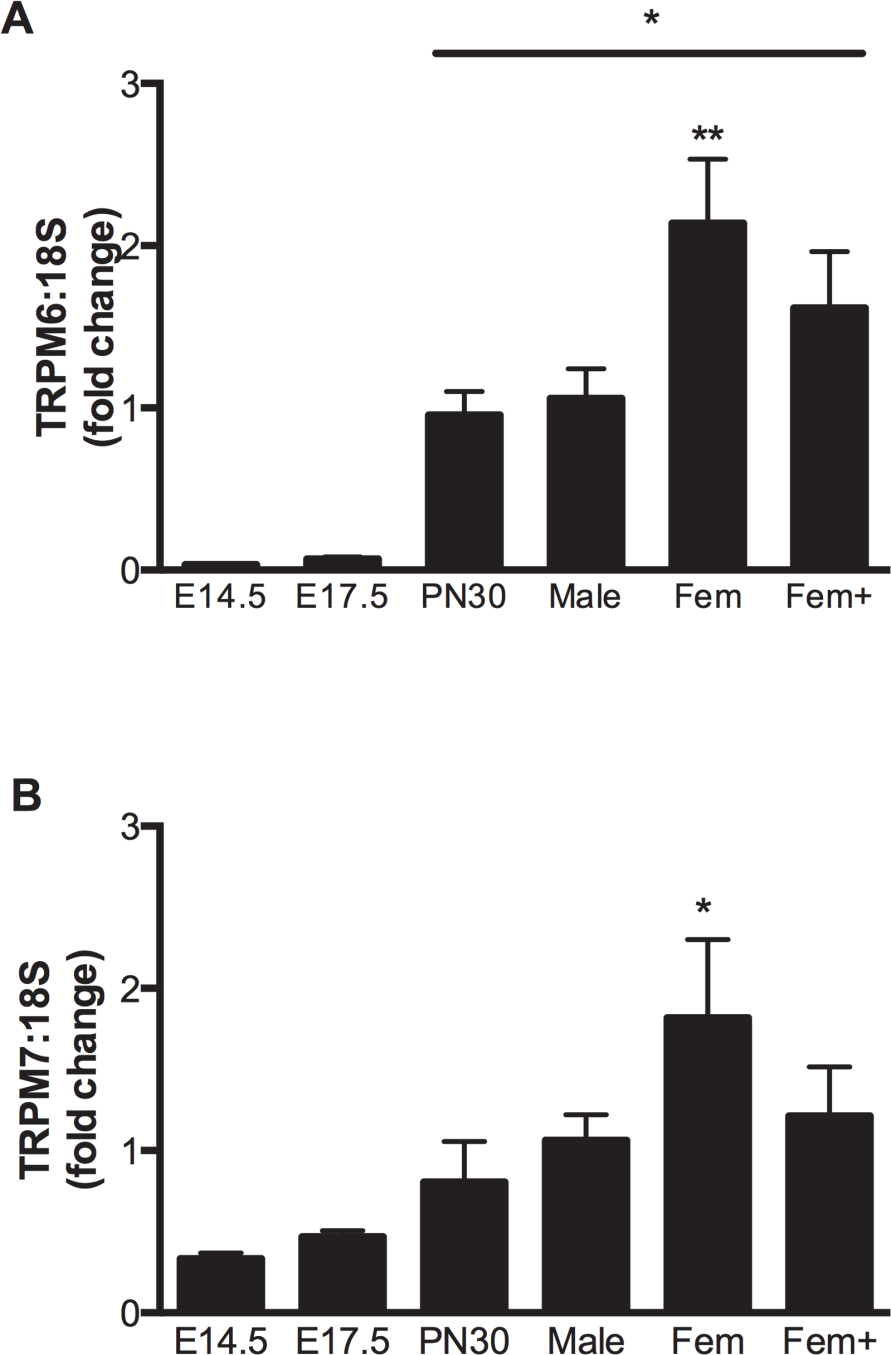
Expression of TRPM6 and TRPM7 mRNA in the mouse kidney during development. Expression of TRPM6 (A) and TRPM7 (B) mRNA (relative to 18S rRNA) in the kidneys of mice at embryonic days 14.5 and 17.5 (E14.5 and E17.5), postnatal day 30 (PN30), and adult male, female (Fem) and pregnant female (Fem+) animals. Data is expressed as mean ± SEM relative to the adult male, n = 5 (E14.5), 5 (E17.5), 9 (PN30), 5 (adult male), 5 (adult female), 5 (pregnant female). * *P*<0.05 vs E14.5 and E17.5; ** *P*<0.05 vs adult male.

TRPM6 and TRPM7 mRNA transcripts were detected in the kidney at all developmental time points examined (S1 Table), with both channels showing specific differences in expression throughout development. In whole kidney homogenates, TRPM6 expression was ∼ 15–30 fold less in fetuses (E14.5 and E17.5) compared to adult males (Fig. 1A, *P*<0.05). In adult mice, TRPM6 showed sex-specific regulation in the kidney, with non-pregnant females having twice the expression of males. This sex-specific increase in renal TRPM6 expression was blunted in pregnant mice (Fig. 1A). In contrast, renal TRPM7 expression remained constant throughout fetal development and early life. In adult mice, renal TRPM7 expression was also increased (∼1.8 fold, *P*<0.05 vs males) in non-pregnant females compared to males, with this increase being absent in pregnant animals (Fig. 1B).

### Cardiac TRPM6/7 expression

**Fig 2:**
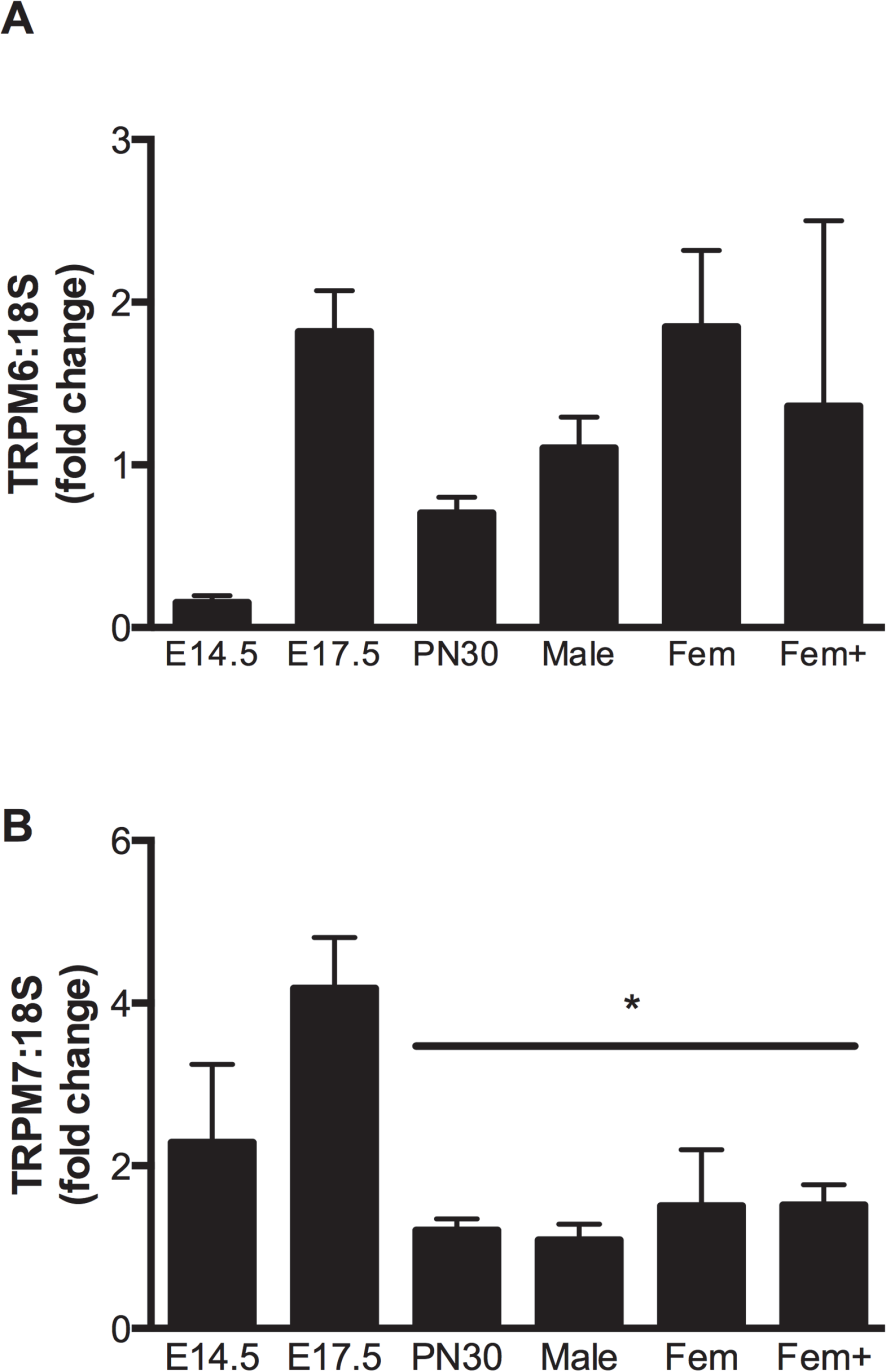
Expression of TRPM6 and TRPM7 mRNA in the mouse heart during development. Cardiac expression of TRPM6 (A) and TRPM7 (B) mRNA (relative to 18S rRNA) at embryonic days 14.5 and 17.5 (E14.5 and E17.5), postnatal day 30 (PN30), as well as in adult male, female (Fem) and pregnant female (Fem+). Data is expressed as mean ± SEM relative to the adult male, n = 5 (E14.5), 6 (E17.5), 9 (PN30), 6 (adult male), 5 (adult female), 4 (pregnant female). * *P*<0.05 vs E17.5.

Surprisingly, TRPM6 expression (as determined by qPCR) was detected in the heart at all developmental time points examined, albeit at much lower levels than in the kidney (S1 Table). Expression in whole heart homogenates increased between E14.5 and E17.5 before decreasing at PN30 (Fig. 2A). TRPM7 was also expressed in the heart throughout development, with the expression levels ∼ 2–4 fold higher in fetal hearts than in adult male hearts (Fig. 2B, *P*<0.05).

### Localization of TRPM6/7 channel expression

**Fig 3:**
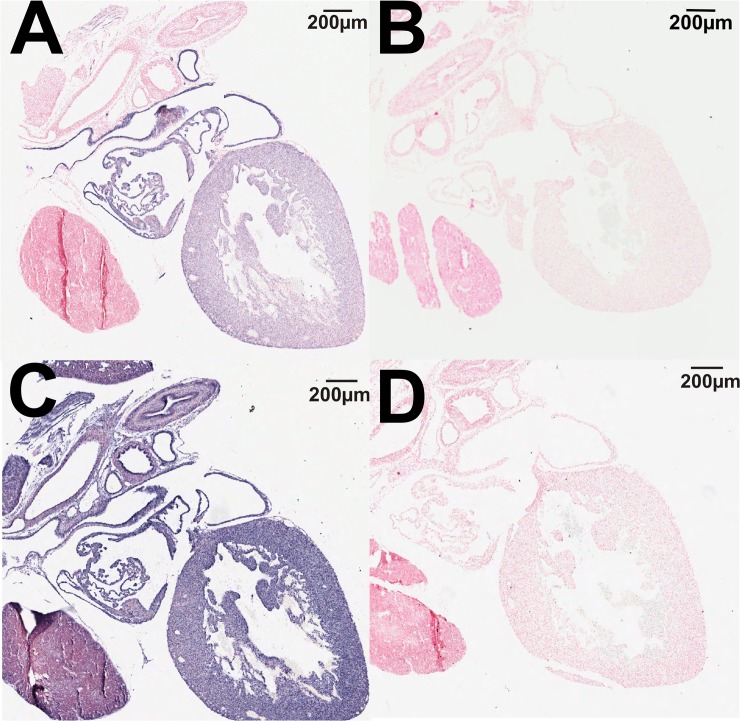
Localization of TRPM6 and TRPM7 mRNA in the fetal heart. *In situ* hybridization using antisense probes showing the localization of TRPM6 (A) and TRPM7 (C) in the fetal heart. Negative controls using the corresponding sense probes showed no staining for TRPM6 (B) or TRPM7 (D). RNA hybridization results in blue staining. All sections were counterstained using nuclear fast red. Scale bars represent 200 μM.

**Fig 4:**
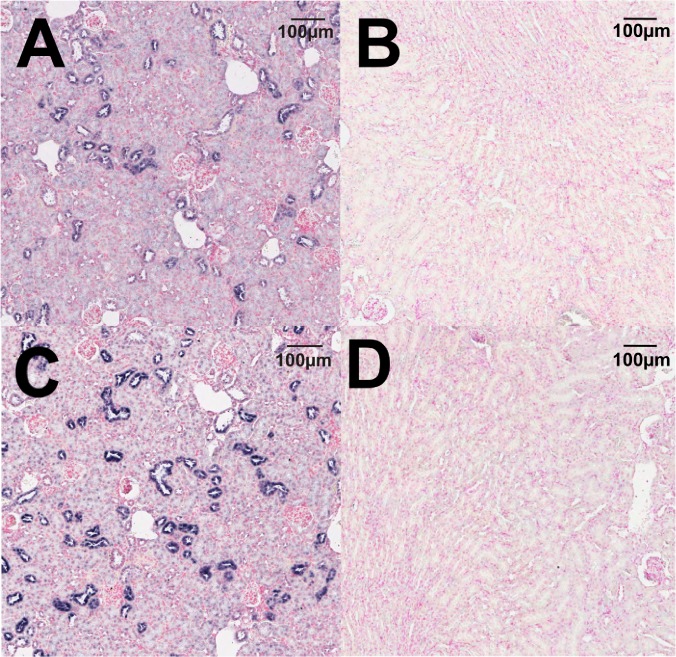
Localization of TRPM6 and TRPM7 mRNA in the adult kidney. *In situ* hybridization using antisense probes showing the localization of TRPM6 (A) and TRPM7 (C) in the adult kidney. Negative controls using the corresponding sense probes showed no staining for TRPM6 (B) or TRPM7 (D) in the adult kidney. RNA hybridization results in blue staining. All sections were counterstained using nuclear fast red. Scale bars represent 100 μM.

*In situ* hybridization studies supported the qPCR results demonstrating that TRPM6 mRNA is expressed in the fetal heart at E17.5 (Fig. 3A). The negative control sense probe showed no straining in the adjacent section (Fig. 3B), indicating the specificity of staining. TRPM7 was ubiquitously expressed throughout the fetal heart (Fig. 3C) with the sense probe demonstrating no staining (Fig. 3D). *In situ* hybridization of TRPM6 in adult kidneys demonstrated the expected tubular localization in the adult kidney (Fig. 4A), whilst the sense probe showed no staining in an adjacent section (Fig. 4B). TRPM7 also demonstrated a tubular localization in the adult kidney (Fig. 4C), and staining specificity was again confirmed by using a sense probe as a negative control (Fig. 4D).

### Regulation of TRPM6/7 channels by glucocorticoids

**Fig 5:**
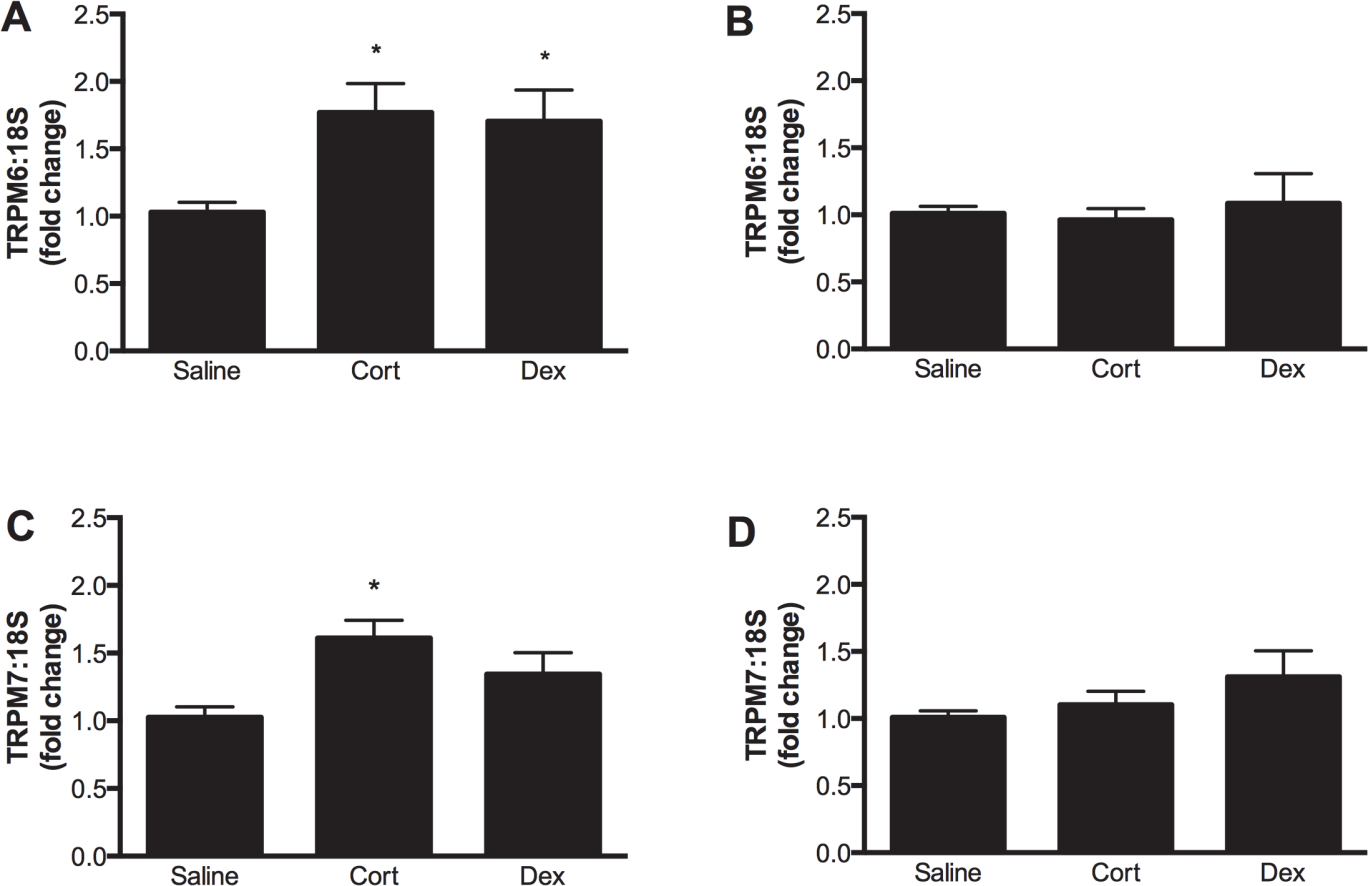
Regulation of TRPM6 and TRPM7 in the fetal heart by glucocorticoids. Expression of TRPM6 (top panels) and TRPM7 (bottom panels) mRNA (relative to 18S rRNA) in the fetal hearts of mice exposed to either corticosterone (Cort) or dexamethasone (Dex) for 60 hours during gestation. In panels A and C, expression was measured at embryonic day 14.5, immediately after glucocorticoid exposure. In panels B and D, expression was measured at embryonic day 17.5, ∼ 60 hours after the cessation of glucocorticoid treatment. Data is expressed as mean ± SEM relative to the saline control, n = 12 (E14.5 saline), 10 (E14.5 Cort), 12 (E14.5), 10 (E17.5 saline), 11 (E17.5 Cort), 4 (E17.5 Dex). * *P*<0.05 vs saline control.

**Fig 6:**
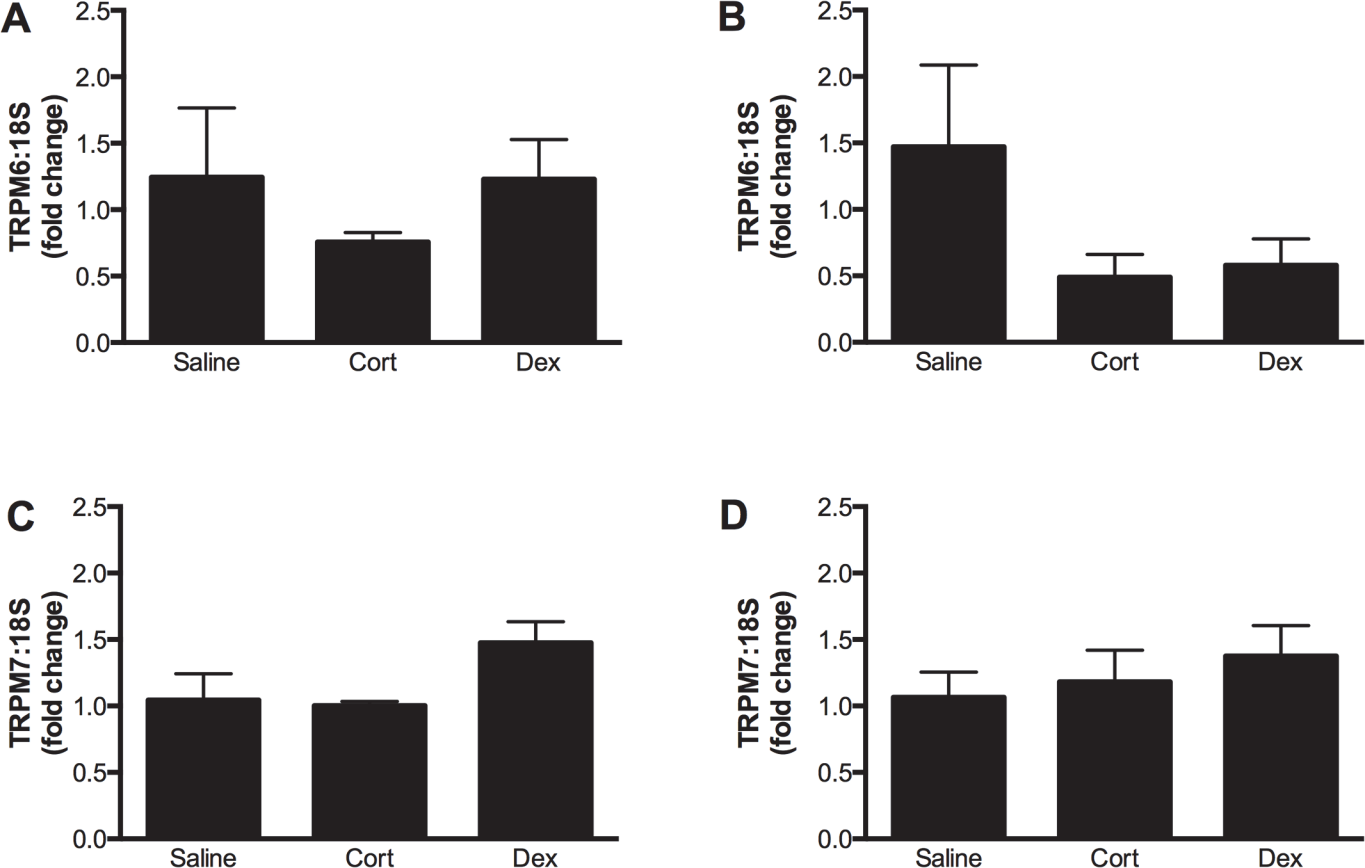
Regulation of TRPM6 and TRPM7 in the maternal heart by glucocorticoids. Expression of TRPM6 mRNA (relative to 18S rRNA) at 14.5 (A) and 17.5 (C) days of gestation, and TRPM7 mRNA at 14.5 (B) and 17.5 (D) days of gestation in the hearts of pregnant mice treated with either corticosterone (Cort) or dexamethasone (Dex) for 60 hours starting at gestational day 12.5. Data is expressed as mean ± SEM relative to the saline control, n = 4 (E14.5 saline), 5 (E14.5 Cort), 5 (E14.5 Dex), 5 (E17.5 saline), 5 (E17.5 Cort), 5 (E17.5 Dex).

The expression of both TRPM6 and TPRM7 mRNA was regulated by maternal glucocorticoid administration in a time- and tissue-dependent manner. In the fetal heart, TRPM6 expression was increased ∼1.7 fold at E14.5 by the administration of either Cort or Dex to the dam (Fig. 5A). A similar trend was seen for TRPM7 expression, although this was only statistically significant following Cort exposure (Fig. 5C). By E17.5 (∼ 60 h after the cessation of glucocorticoid treatment) fetal cardiac TRPM6 and TRPM7 expression had returned to the level of the saline control (Fig. 5 B & D). In contrast to the effects on the fetal heart, administration of glucocorticoids did not alter TRPM6 and TRPM7 mRNA levels in the maternal heart (Fig. 6).

**Fig 7:**
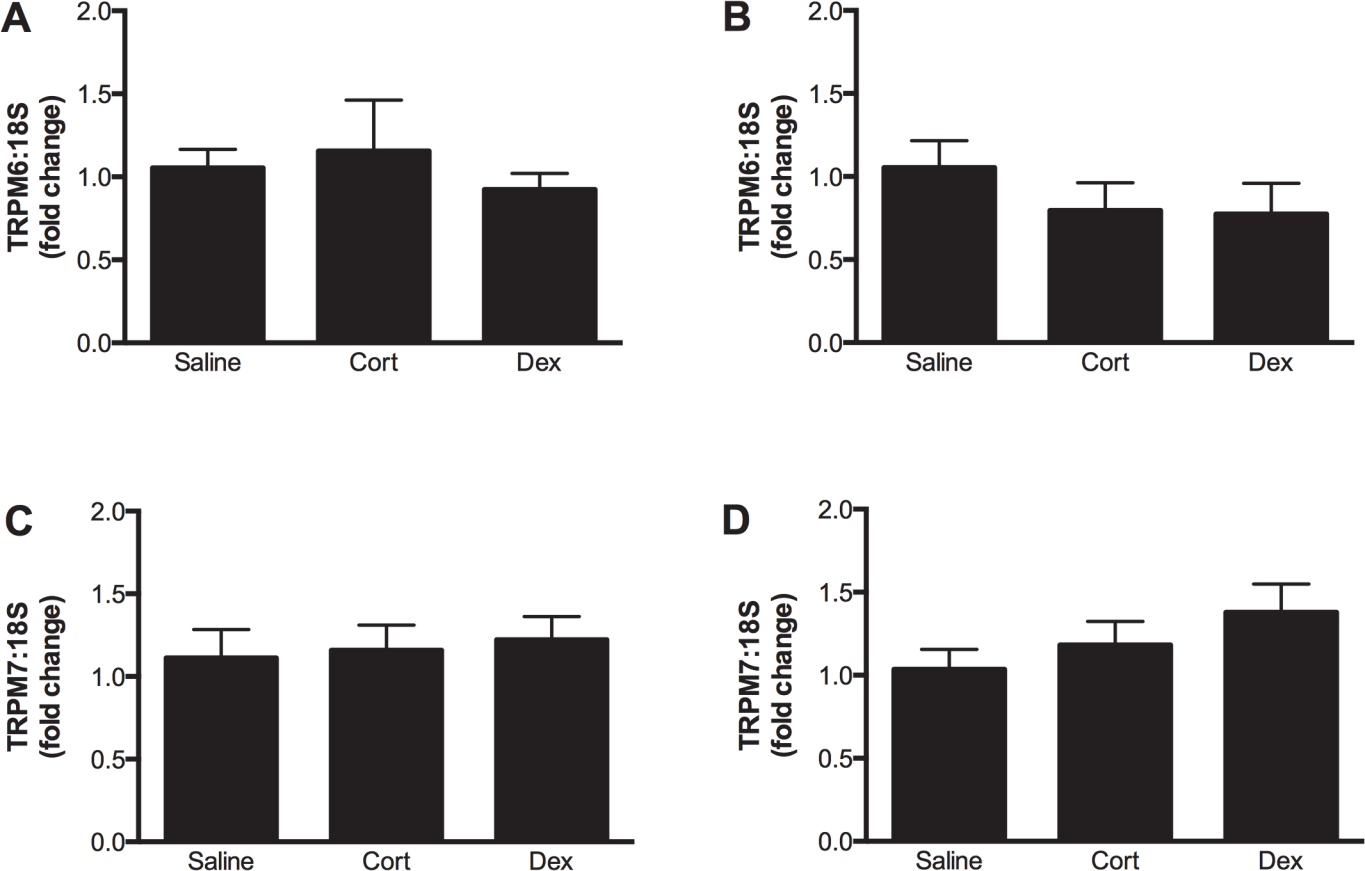
Regulation of TRPM6 and TRPM7 in the fetal kidney by glucocorticoids. Expression of TRPM6 (top panels) and TRPM7 (bottom panels) mRNA (relative to 18S rRNA) in the fetal kidneys of mice exposed to either corticosterone (Cort) or dexamethasone (Dex) for 60 hours during gestation. In panels A and C, expression was measured at embryonic day14.5, immediately after glucocorticoid exposure. In panels B and D, expression was measured at embryonic day 17.5, ∼ 60 hours after the cessation of glucocorticoid treatment. Data is expressed as mean ± SEM relative to the saline control, n = 11 (E14.5 saline), 8 (E14.5 Cort), 6 (E14.5 Dex), 6 (E17.5 saline), 6 (E17.5 Cort), 6 (E17.5 Dex).

**Fig 8:**
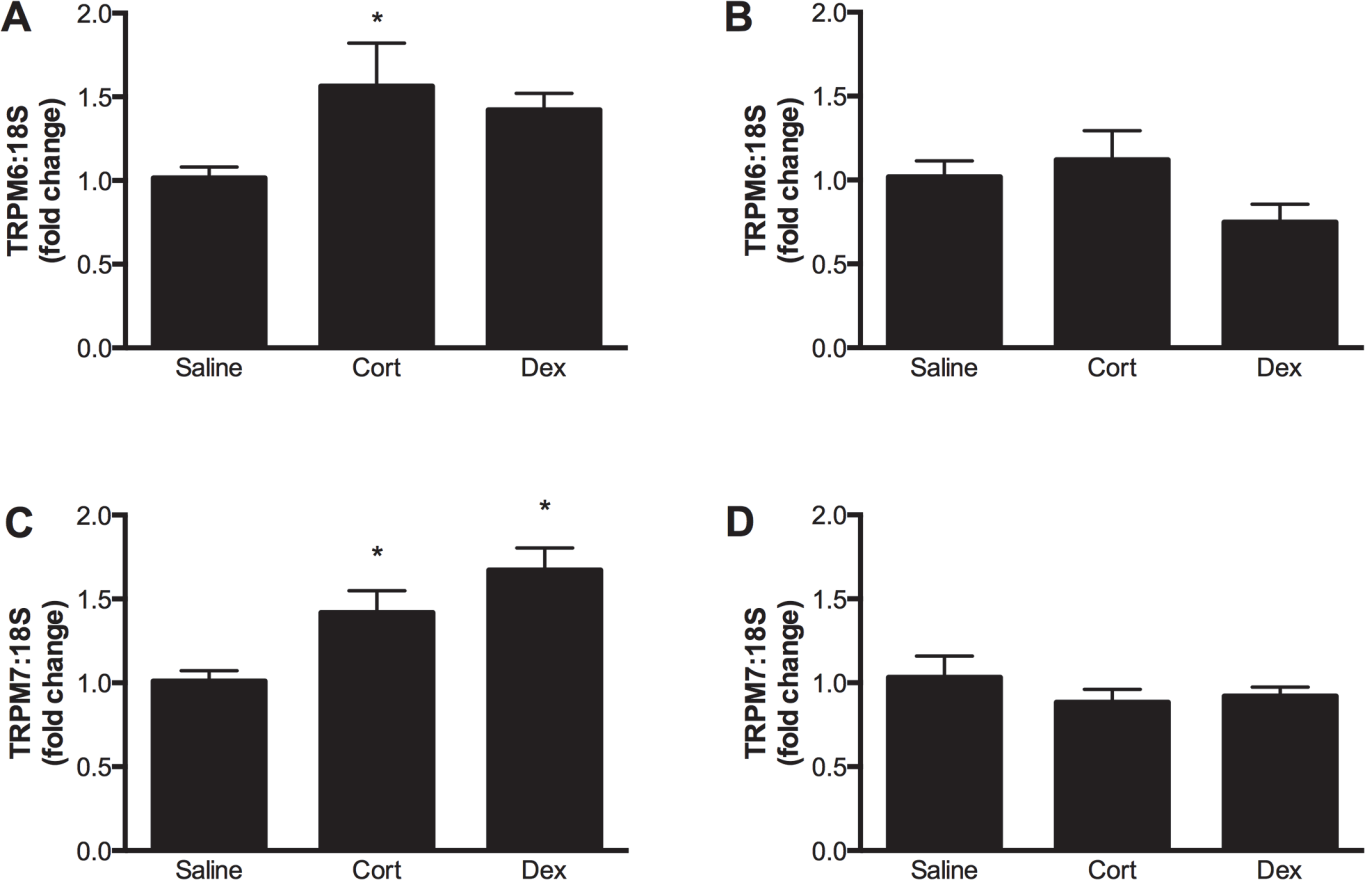
Regulation of TRPM6 and TRPM7 in the maternal kidney by glucocorticoids. Expression of TRPM6 mRNA (relative to 18S rRNA) at 14.5 (A) and 17.5 (C) days of gestation, and TRPM7 mRNA at 14.5 (B) and 17.5 (D) days of gestation in the kidneys of pregnant mice treated with either corticosterone (Cort) or dexamethasone (Dex) for 60 hours starting at gestational day 12.5. Data is expressed as mean ± SEM relative to the saline control, n = 7 (E14.5 saline), 5 (E14.5 Cort), 5 (E14.5 Dex), 5 (E17.5 saline 5), 5 (E17.5 Cort), 5 (E17.5 Dex).

Maternal glucocorticoid administration had no effect on the expression of TRPM6 or TRPM7 in the fetal kidney at E14.5 (Fig. 7), but increased TRPM6 and TRPM7 expression in the maternal kidney at this time (Fig. 8A & C). Maternal and fetal renal expression of TRPM6 and TRPM7 were similar in all groups at E17.5, ∼ 60 h after the end of glucocorticoid treatment (Fig. 8B & D). Despite these changes in magnesium channel expression, glucocorticoid treatment caused no significant changes in plasma electrolytes in the dam (Mg^2+^ plasma concentrations in mmol/L: saline 0.69±0.06; corticosterone 0.72±0.08; dexamethasone 0.71±0.05).

## Discussion

This study is the first to demonstrate that TRPM6 and TRPM7 are differentially expressed in the heart and kidney throughout gestation and into adulthood. We have uniquely identified mRNA for TRPM6 (traditionally considered to be an epithelial channel) in the fetal mouse heart, with expression levels being higher at E17.5 than in PN30 offspring. Importantly, we showed that administration of glucocorticoids during pregnancy can regulate TRPM6 and TRPM7 expression in a time- and tissue-dependent fashion in both the dam and fetus. When considered in context of recent findings suggesting a critical role for TRPM channels in organogenesis, these data suggest a novel mechanism by which maternal glucocorticoid exposure may influence the developing fetus. This is of particular interest as we have shown previously that maternal exposure to either corticosterone or dexamethasone impairs placental development and fetal growth [23,24], and that our model of maternal dexamethasone exposure impairs cardiac growth, reduces nephron number and elicits long term functional alterations in blood pressure and pulse pressure [25] TRPM6 was first identified in human studies of inherited hypomagnesaemia, and shown to be predominantly expressed in intestinal and renal epithelia [4]. It plays a major role in maintaining Mg homeostasis in the adult by controlling the active absorption of Mg from the gut and reabsorption of filtered Mg in the kidney [2]. Despite the importance of TRPM6 in magnesium handling, little is known about how TRPM6 expression and function changes throughout development and ageing. Here we show for the first time that renal TRPM6 is developmentally regulated, with very low levels of expression *in utero* that increase after birth. This is consistent with the idea that whilst TRPM6 is important for magnesium homeostasis in the adult animal, in the fetus magnesium homeostasis is largely regulated by maternal magnesium status and transport across the placenta [28] and there is little need for fetal renal magnesium reabsorption.

Surprisingly, we also detected expression of TRPM6 in the fetal heart, albeit at low levels compared to that of the adult kidney. We confirmed the qPCR data using *in situ* hybridization, and detected robust expression of TRPM6 throughout the fetal heart. The functional significance of TRPM6 expression in the fetal heart remains unknown. One possibility is that TRPM6 expression in the fetal heart is modifying the function of TRPM7 via TRPM6/7 complex formation. TRPM6 and TRPM7 have been shown to form hetrotetrameric channel complexes that have unique electrophysiological characteristics compared to homomeric channels [29,30]. Chubanov et al. demonstrated that TRPM6/TRPM7 heterooligomerization was essential for forming a functional TRPM6-containing channel complex in the membrane of epithelial cells [31], suggesting that interactions between TRPM6 and TRPM7 have an important biological role. TRPM7 is critical for early cardiogenesis [32], and it is possible that early myocardial development may be influenced by both homotetrameric TRPM7 channels and heteromeric TRPM6/7 channel complexes. Alternatively, TRPM6 may be important in development for reasons unrelated to TRPM7. Studies in transgenic animals show that TRPM6 deficiency is embryonically lethal, with most pups dying before E12.5 [16,33]. Animals that survived to term often displayed neural tube defects, a surprising finding given that studies in the adult support a primarily intestinal and renal role of TRPM6 [16]. Dietary supplementation of magnesium to the dams did not rescue the TRPM6-/- mice [16,33], suggesting that the importance of TRPM6 in development may extend beyond magnesium homeostasis. As these studies have involved global deletion of TRPM6, determining the functional significance of TRPM6 in the development of individual organs will require the use of transgenic animals with tissue-specific deletion of this channel. It is important to note that there are differences between the phenotype of TRPM6-/- mice and patients with loss-of-function TRPM6 mutations. In humans, mutations in the TRPM6 gene lead to hypomagnesaemia with secondary hypocalcaemia (HSH), an autosomal recessive condition characterized by excessive renal magnesium wasting [4,5]. The electrolyte abnormalities lead to neurological symptoms (e.g. seizures, neuromuscular abnormalities) that manifest in infancy and can be controlled with prompt initiation of magnesium therapy [34]. The contrast between the clinical presentation of TRPM6 mutation in humans and the lethal phenotype of TRPM6-/- mice suggests that the physiological and developmental roles of TRPM6 are yet to be fully elucidated.

The pattern of TRPM7 expression in the heart throughout development mirrored that of TRPM6: both were more highly expressed in the fetus than in the adult. However, the difference in expression levels was much more pronounced for TRPM6 than for TRPM7 (15–30 fold vs 2–4 fold higher in the fetal heart compared to the adult). High levels of TRPM7 expression in the fetal heart have been previously reported by Jin et al, who showed that at E9.5 expression of TRPM7 in the mouse was predominantly localized to the heart before becoming more ubiquitously expressed throughout the fetus from E11.5 − 14.5 [15]. We have used a quantitative method to show that expression of TRPM7 in the mouse heart remains high until late gestation, before declining after birth and remaining relatively constant in adulthood. A number of recent studies have identified a critical but complex role for TRPM7 in cardiac development and function. Cardiac-specific deletion of TRPM7 in early cardiogenesis (prior to E9) is embryonic lethal, whilst embryos with cardiac deletion of TRPM7 at a later time point (after E12.5) survived to adulthood with no overt alterations in basal cardiac function [32]. In the adult, TRPM7 in the sinoatrial node is required to maintain automaticity [35], and its importance in cardiac electrophysiology is further highlighted by work showing upregulation of TRPM7 in fibroblasts from patients with atrial fibrillation, an effect that may contribute to fibrogenesis [36]. Thus, TRPM7 appears to be a critically important protein for cardiac function, making it important to understand the potential factors, such as sex and age, which may regulate and/or disrupt expression of this protein.

In contrast to the marked upregulation of renal TRPM6 that occurs after birth, expression of TRPM7 in the kidney remains relatively constant throughout fetal development and adulthood. This suggests that TRPM7 may play a more prominent role during renal development than TRPM6, contributing to developmental processes such as cell proliferation. Indeed, tissue specific knockouts have shown that TRPM7 is important in nephrogenesis, as deletion of TRPM7 in the metanephric mesenchyme reduced the number of glomeruli formed *in utero* [37]. Whilst few studies have directly examined the functional importance of TRPM7 in the adult kidney, it is known that factors such as chronic aldosterone administration [17] and ischemia-reperfusion injury [38] can increase renal TRPM7 mRNA levels, suggesting that this protein may also be important for renal health in the adult.

Interestingly, in the adult kidney both TRPM6 and TRPM7 exhibited sex-specific expression differences. In the adult kidney TRPM6 expression was ∼ 2-fold higher in females than in males. This is consistent with previous work demonstrating that ovariectomy reduces renal TRPM6 expression by ∼ 50% [6]. This ovariectomy-induced reduction in renal TRPM6 was normalized by administration of exogenous 17β-estradiol, thus demonstrating that estrogen can regulate TRPM6 expression. In addition to these classic transcriptional effects estrogen can also influence the TRPM6 channel via non-transcriptional mechanisms, as treating TRPM6-expressing cells with 17β-estradiol rapidly increases TRPM6-mediated Mg^2+^ influx [39]. Despite the documented role of estrogen in regulating renal TRPM6 expression, the current study found that pregnancy had no effect on renal TRPM6 expression despite the substantial increase in estrogen levels that occurs during pregnancy. This suggests that during pregnancy, TRPM6 regulation may be under the control of different mechanisms. It is important to note although estrogen is known to increase TRPM6 expression, plasma estrogen and magnesium levels are inversely related both in women of reproductive age [40] and in menopausal women[41]. Interestingly, while some studies have reported lower plasma magnesium levels in pregnancy [42], a recent longitudinal study in pregnant women reported that serum magnesium levels were similar in all three trimesters, despite the large increase in plasma estrogen that occurs in the third trimester [43]. This suggests that there are additional mechanisms contributing to the regulation of TRPM6 and magnesium homeostasis during pregnancy to maintain physiologically normal levels of magnesium despite the large hormonal changes that occur in pregnancy. In this study, we also found that non-pregnant adult females had higher levels of renal TRPM7 expression than adult males. This sex-specific expression was an unexpected finding, as a previous study in rats showed no alterations in TRPM7 mRNA levels with ovariectomy or exogenous 17β-estradiol [6]. This further suggests that the sexual disparity in renal TRPM7 expression in mice seen in the present study is not directly related to estrogen levels.

After establishing the ontogeny of TRPM6 and TRPM7 expression in the heart and kidneys, we then examined how maternal administration of glucocorticoids affected these channels during pregnancy. Maternal exposure to both natural and synthetic glucocorticoids is common in human pregnancy [20], and is associated with adverse long-term outcomes [44]. Glucocorticoids have been shown to affect the expression of TRPM channels in non-pregnant adults but the effects of administration during pregnancy are unknown. In the maternal kidney, mRNA levels of TRPM7 were increased by both corticosterone and dexamethasone, whilst corticosterone also increased TRPM6 mRNA. These changes were temporary, as channel expression had returned to control levels by 60 h after the end of glucocorticoid treatment. The glucocorticoid-induced changes in channel expression in the dam were organ-specific, as expression of TPRM6 and TRPM7 in the heart were unaltered by either dexamethasone or corticosterone. Renal-specific alterations in TRPM6 expression following dexamethasone treatment have been reported previously in male rats [19]. At present, it is not known whether organ-specific alterations in channel expression are due to the direct versus indirect effects of glucocorticoids, or tissue-specific differences in glucocorticoid metabolism and receptor signaling. The present study suggests that regulation of renal expression of TRPM6 and TRPM7 by glucocorticoids also occurs in pregnant females.

We have shown that short term maternal glucocorticoid exposure alters fetal renal and cardiac development during pregnancy and predisposes offspring to disease in adulthood [25,45,46] In fact, in the same model of dexamethasone exposure as the current study we found impaired fetal heart growth, reduced nephron endowment and increased pulse pressure in male offspring [25]. In the current study TRPM channels in the fetal kidney were unaffected by glucocorticoids. The ontogeny data showed TRPM6 levels to be low in the fetal kidney when fetal magnesium homeostasis is largely regulated by maternal magnesium. Thus, it is perhaps unsurprising that whilst glucocorticoids influence renal magnesium reabsorption in the adult, they do not affect TRPM6 expression in the fetal kidney. TRPM7 has been shown to be an important mediator of nephron formation [37], however we found that TRPM7 expression was not affected by glucocorticoid exposure. Thus, in the current study, factors unrelated to TRPM channel expression are likely to be responsible for the glucocorticoid induced nephron deficits previously reported. In contrast, glucocorticoids increased TRPM6 and TRPM7 mRNA levels in the fetal heart at E14.5. These glucocorticoid induced changes in TRPM channel expression may be important considering that both TRPM6 and TRPM7 are highly expressed during fetal cardiac development. Although the consequences of glucocorticoid induced alterations in cardiac TRPM channel expression during development are unknown, they may influence the expression of cardiac growth factors. We have previously reported that dexamethasone increases cardiac expression of IGF-1[25], a hormone which itself is involved in cellular magnesium metabolism [47]. Thus, it is tempting to speculate that the previously reported changes in heart formation may be linked to the altered expression of these channels.

One limitation of the present study is the absence of data showing changes in the protein expression of TRPM6 and TRPM7. Despite numerous attempts we have been unable to use commercially available antibodies to quantify changes in TRPM6/7 protein by Western blotting, as none of the antibodies we tested showed specificity for the target protein. The ability of some commercial antibodies to demonstrate nonspecific binding, particularly in tissue homogenates, is a cause of concern that is limiting the progress of a number of fields. Here, whilst we assume that protein expression of TRPM6 and TRPM7 will parallel our qPCR and *in situ* hybridization data, the lack of reliable tools means that we cannot provide direct evidence for this.

The present study describes an mRNA expression profile for TRPM6 and TRPM7 that is both developmentally regulated and affected by maternal glucocorticoid administration. These spatiotemporal alterations in expression profiles support previous work showing that these channels are important for normal fetal organogenesis, particularly of the heart. Furthermore, we show that maternal exposure to elevated glucocorticoid levels can directly alter expression of these channels in the developing fetal heart. Consequently, maternal perturbations that disrupt the normal hormonal systems associated with pregnancy may influence fetal magnesium exposure and the development of the growing fetus.

## Supporting Information

S1 Table Expression levels of TRPM6 and TRPM7 mRNA in the mouse heart and kidney throughout development. (DOCX)
