# Supplementary material for: Differential mRNA Expression and Glucocorticoid-Mediated Regulation of TRPM6 and TRPM7 in the Heart and Kidney throughout Murine Pregnancy and Development
Source: PLoS One. 2015 Feb 18;10(2):e0117978. doi: 10.1371/journal.pone.0117978 (PMC4333289; doi:10.1371/journal.pone.0117978)
Supplement: S1 Table — (DOCX) [file pone.0117978.s001.docx]

**Tables**

S1 Table. Expression levels of TRPM6 and TRPM7 mRNA in the mouse heart and kidney throughout development

|  |  | E14.5 | E17.5 | PN30 | Male  Adult | Female Adult | Female pregnant |
| --- | --- | --- | --- | --- | --- | --- | --- |
| TRPM6 (∆CT) | Heart | 21.98±0.41 | 18.26±0.20 | 19.66±0.19 | 19.05±0.31 | 18.43±0.46 | 20.21±1.18 |
|  | Kidney | 18.89±0.16 | 17.94±0.11 | 14.17±0.17 | 14.01±0.23 | 13.03±0.30 | 13.47±0.34 |
| TRPM7 (∆CT) | Heart | 14.62±0.51 | 13.68±0.32 | 15.23±0.18 | 15.42±0.27 | 14.90±0.26 | 15.46±0.87 |
|  | Kidney | 9.64±0.17 | 9.67±0.19 | 8.73±0.40 | 8.02±0.27 | 7.38±0.41 | 7.92±0.36 |

E-embryonic day, PN-postnatal day, ∆*CT*- Cycle threshold value (CT) of TRPM6/7 – CT of 18s. The expression of 18s was similar at all developmental time points (CT 12 – 14), and data is shown as mean ± SEM.
